# Dietary and serum antioxidant capacity is inversely associated with patients in osteoarthritis: a case-control study

**DOI:** 10.1186/s41043-024-00566-8

**Published:** 2024-07-04

**Authors:** Beda Büşra Özalp Çolak, Nilgün Seremet Kürklü, Kübra Tel Adıgüzel, Emre Adıgüzel

**Affiliations:** 1 Department of Nutrition and Dietetics, Faculty of Health Sciences, Yüksek İhtisas University, Ankara, Turkey; 2 Department of Nutrition and Dietetics, Faculty of Health Sciences, Akdeniz University, Antalya, Turkey; 3 Department of Nutrition and Dietetics, Gülhane Faculty of Health Sciences, University of Health Sciences Turkey, Ankara, Turkey; 4 Physical Medicine and Rehabilitation Hospital, Ankara Bilkent City Hospital, Ankara, Turkey

**Keywords:** Antioxidants, Nutrition status, Osteoarthritis

## Abstract

This study aimed to examine dietary antioxidant and serum antioxidant capacity in patients with knee osteoarthritis (OA). This case-control study consisted of 47 patients with OA (case group) and 30 healthy subjects (control group). The control and case group were matched age, gender, and body mass index (*p* > 0.05). A food frequency questionnaire was administered to participants, and dietary total antioxidant capacity (DTAC) was estimated using the ferric reducing antioxidant power method (FRAP). Participants’ serum total antioxidant capacity (TAC) and total oxidant capacity (TOC) measurements were performed, and the oxidative stress index (OSI) was calculated. DTAC of case group was found to be lower than the control group (*p* < 0.05). The daily consumption of red meat and butter of the individuals in the case group was higher than that of the control group, and their fish consumption, dietary vitamin A and carotene intakes were found to be lower (*p* < 0.05). In addition, OA patients have TAC and OSI was also found to be significantly higher than in control group (*p* = 0.001 and *p* < 0.001). Since low dietary total antioxidant capacity and high serum total oxidant capacity, individuals with OA should pay more attention to their diet to increase serum antioxidant status.

## Introduction

Osteoarthritis (OA) is one of the most seen types of arthritis and the leading causes of movement limitations. Approximately 16% of the global population has knee OA, and these individuals cannot perform routine daily activities and reduced quality of life [1–3].

Osteoarthritis is a multifactorial inflammatory bone-joint disease, including deterioration of the subchondral bone structure and cartilage degeneration. Inflammatory cytokines have a negative effect on disease progression by activating catabolic pathways. In addition to inducing oxidative stress and low-level systemic inflammation, obesity causes OA by increasing the load applied to the knee joints [4]. Several studies have reported that changes to dietary habits and lifestyle in slightly overweight and obese individuals with OA can be effective in decreasing the symptoms and complications of OA [4–6].

Several nutrients or foods in the diet of individuals with OA are extremely effective in respect of OA treatment. Previous studies have shown that a diet rich in dairy products, fish, fibre such as fruit and vegetables, omega-3 fatty acids, and antioxidant vitamins and minerals, reduces the risk of OA and is effective in treatment [7, 8].

Total antioxidant capacity and total oxidant capacity are defined as the cumulative total of all the antioxidants in foodstuffs or body fluids. A shift towards oxidants in the antioxidant-oxidant balance creates oxidative stress. Some recent studies have shown that increased free oxygen radicals and lipid peroxidation play a role in the etiology of several diseases [4, 9]. Total antioxidant capacity values are measured to evaluate in vivo antioxidants in biological fluids and the nutritional status. This capacity is affected by an antioxidant-rich diet and the consumption of antioxidant supplements. Cellular antioxidant capacity mainly contributes to the antioxidant enzyme system, and diet-origin antioxidants affect the plasma to a large degree [10, 11].

Oxidative stress, which causes an increase in reactive oxygen species (ROS), has an important role in the formation and progression of OA. Free radicals, ROS, and various derivatives accumulate in the synovial joints in OA, and associated with this, cell death and inflammation are observed. Therefore, there is a view that the formation and progression of OA can be prevented with the consumption of antioxidant nutrients [12, 13]. Synovial inflammation plays a role in both the development and progression of osteoarthritis [14]. Daneshzad et al. [15] examined that dietary antioxidant and adipokines level among obese women and found some adipokines levels directly associated with dietary vitamins D and A intake. Similar study was observed link between obesity and DTAC in healthy women. The study found that DTAC were significantly positively associated with obesity [16]. Nutrional markers are reliable disease markers inflamation, such as type 2 diabetes [17], and survival of geriatric patients [18]. To the best of our knowledge, there is a limited number of studies in literature that have examined the relationship between total antioxidant capacity of the diet and osteoarthritis.

Therefore, the aim of this study was to evaluate the nutritional status, dietary antioxidant intake, and serum total antioxidant/oxidant capacity of OA patients.

## Materials and methods

### Study sample

This case-control, descriptive study was conducted to evaluate the nutritional status and dietary total antioxidant capacity and serum total antioxidant/oxidant capacity of patients diagnosed with OA (Kellgren-Lawrence Grade ≥ II). Sampling was done by using Vanderbilt University Sample Calculation Tool [19] and benefiting from antioxidant capacity studies [20, 21] previously conducted in individuals with osteoarthritis. Using independent design t-test with the inputs for α 0.05, power 0.80, for 𝛿 0.12, and for σ 0.20, it is calculated that at least 45 patients were required in the sample. The study included 47 patients, aged 18–64 years, who were diagnosed with OA at a tertiary level healthcare institution between April 2020 and February 2022. Since aging may alter antioxidant capacity, people aged 65 years and older were not included in the study. These patients constituting the case group had no chronic disease, did not smoke or drink alcohol, were not using corticosteroids, and had not taken vitamin or mineral supplements within the last 6 months. A control group was formed of 30 healthy individuals who presented at the same polyclinic were not diagnosed with OA and met the same criteria.

The standing bidirectional knee radiographs taken during the diagnosis were interpreted by an experienced physiatrist according to the Kellgren Lawrence scale and osteoarthritis staging was recorded [22].

The protocol was reviewed and approved by the Ethics Committee of Akdeniz University (code 70,904,504/659). All procedures followed the guidelines of the Helsinki Declaration, and the study was registered at Clinical Trials (ID-NCT05214469).

### Questionnaire survey

The questionnaire applied to all the participants included descriptive information in the first section (age, gender, education level). Nutritional habits were then questioned. To determine the physical activity level of the participants, the International Physical Activity Questionnaire (IPAQ)-Short Form was administered [23]. Body mass index (BMI), which is important in determining and evaluating body weight was calculated as body weight (kg)/ height (m^2^) and evaluated according the World Health Organization (WHO) classification [24].

To be able to correctly interpret the study results, care was taken in the selection of the sample that the individuals in the case and control groups were similar in respect of age, gender, and BMI (*p* > 0.05).

A pre-prepared food consumption form was used with the 24-hour recall method to determine nutrient intake [25]. The data obtained from the food intake records were analyzed with the Nutritional Information Systems Package program using Standard Food Recipes [26]. The daily energy nutrient component intakes and amounts of food groups consumption were calculated.

### Dietary total antioxidant capacity (DTAC)

A food frequency (and amount) consumption form was applied to all the study participants to evaluate nutrient consumption. The food consumption frequency was evaluated based on the food consumption of the individual within the last year. The dietary total antioxidant capacity was calculated through a validated 229-item food frequency questionnaire [27]. The dietary total antioxidant capacity was calculated from the amounts on the food frequency consumption form. The dietary total antioxidant capacity was calculated using the antioxidant database of foods created in 2010 by Carlsen et al. with the FRAP method [11].

### Serum total oxidant and antioxidant capacity (TOC, TAC)

The TAC level and total oxidative stress (TOS) measurements were made using the Erel’s method. The serum TAC was measured with the fully automatic method developed by Erel. The Fe dianisidine complex forms a Fenton-type reaction with hydrogen peroxide to form the hydroxide radical. This powerful reactive oxygen type creates yellow brown dianisidyl radicals by entering reaction with the colorless o-dianisidine molecule at a reduced low pH. The color is intensified by the dianisidyl radicals participating in further oxidation reactions. However, antioxidants in the sample halt the color formation by suppressing these oxidation reactions [28]. The result is obtained by measuring this reaction spectrophotometrically on the automatic analyzer. To measure TAC and TOC, 2 mL venous blood was withdrawn from the forearm inner surface (the normal region for taking blood samples). After centrifugation of the sample at + 4 °C and 3600 rpm, the serum was separated, and this was stored at -80 °C until analysis. The oxidative stress index (OSI) is presented as the quotient of TOS to TAC and expressed in %.

### Statistical analysis

Data obtained in the study were analyzed statistically using SPSS version 22.0 (SPSS Inc., Chicago, IL, USA) software. Conformity of the variables to normal distribution was assessed with the Shapiro Wilk test. In the comparisons between the groups of variables with normal distribution, the Student’s t-test was used, and for variables not showing normal distribution, the Mann Whitney U-test. Results were stated as mean ± standard deviation (SD) values or number (n) and percentage (%). A value of *p* < 0.05 was accepted as statistically significant.

## Results

The general characteristics and nutritional habits of the study participants are shown in Table 1. The case group of OA patients comprised 34 females and 13 males with a mean age of 57.9 ± 9.24 years and the control group included 23 females and 7 males with a mean age of 56.1 ± 10.23 years (*p* > 0.05). The BMI values were determined as mean 31.6 ± 5.92 kg/m^2^ in the OA patients and 30.9 ± 4.23 kg/m^2^ in the control group (*p* > 0.05). The majority (83%) of OA patients were obese or overweight. The groups were similar in respect of the frequency of the number of main meals and the control group were found to have more snacks (*p* < 0.05).

**Table 1:** General characteristics and nutritional habits of the groups

	Case (*n* = 47)		Control (*n* = 30)				*p*	
**Age (years)**	57.9 ± 9.25			56.1 ± 0.23		0.425^a^		
**Male/Female (n)**	13/34		7/23				0.792^a^	
**Radiographic OA severities (n, %)**								
KL = 2	27 (57.5)		-					
KL = 3	16 (34.0)		-					
KL = 4	4 (8.5)		-					
**BMI (kg/m** ^**2**^ **)**	31.6 ± 5.92		30.9 ± 4.23				0.577^a^	
**Overweight/obesity (n, %)**	39 (83.0)		29 (96.7)				0.429^a^	
**METS score (min/wk)** **(Mean ± SD)**	459.9 ± 946.82		330.4 ± 480.80				0.503^a^	
**Number of main meals**								
2 (n, %)	26	55.3	17		56.6		0.900^a^	
3 (n, %)	21	44.7	13		44.4			
**Number of main meals** **(Mean ± SD)**	2.4 ± 0.50		2.4 ± 0.50				0.910^a^	
Number of snacks*								
No snacks	15	32.0	-		-		0.004^a^	
1	13	27.0	12		40.0			
≥ 2	18	41.0	18		60.0			
**Number of snacks (Mean ± SD)**	1.1 ± 0.89		1.7 ± 0.65				**0.002** ^**b**^	
**Missing main meals**								
No	13	27.6	6		20.0		0.498^a^	
Yes	25	53.2	15		50.0			
Sometimes	9	19.2	9		30.0			

^a^ Pearson Chi-square, ^b^ Student’s t-test

KL: Kellgren Lawrence scale

BMI: Body Mass Index

Significant correlations and the corresponding p-values are presented in bold

The mean values of daily energy and the amounts of macro and micronutrients of the groups are shown in Table 2. The energy, carbohydrate protein, fat, omega-6/omega-3 cholesterol, and saturated fatty acids intake were determined to be higher, and fibre omega-3, and vitamins C, E and K intakes were lower in the case group (*p* > 0.05). The amounts of vitamin A (1093.3 ± 749.66 µg) and carotene (4.2 ± 4.41 mg) consumed in the diet were determined to be statistically significantly lower in the OA patient group than in the control group (1907.5 ± 2128.40 µg and 9.3 ± 12.9 mg, respectively) (*p* < 0.05).

**Table 2:** The mean values of daily energy and the amounts of macro and micronutrient of the groups

	Case (*n* = 47)	Control (*n* = 30)			
	Mean ± SD	Mean ± SD	*p*		
Energy (kcal)	1576.5 ± 496.62	1483.5 ± 382.3		0.385^a^	
Carbohydrate (g)	179.4 ± 71.19	171.5 ± 62.43		0.621^a^	
Carbohydrate (E%)	46.1 ± 10.22	46.4 ± 11.23		0.909^a^	
Protein (g)	61.7 ± 18.46	57.8 ± 22.99		0.404^a^	
Protein (E%)	16.5 ± 4.22	16.0 ± 4.02		0.577^a^	
Fat (g)	65.7 ± 24.22	60.4 ± 21.24		0.334^a^	
Fat (E%)	37.6 ± 8.43	37.6 ± 10.60		0.863^a^	
Fibre (total) (g)	21.0 ± 8.3	23.3 ± 10.28		0.276^a^	
Saturated fatty acids (g)	26.3 ± 10.48	22.5 ± 9.27		0.112^a^	
Mono-unsaturated fatty acids (g)	23.6 ± 8.94	22.4 ± 8.72		0.568^a^	
Polyunsaturated fatty acids (g)	10.8 ± 6.68	11.1 ± 5.52		0.823^a^	
Omega 3 (g)	1.3 ± 0.68	1.5 ± 0.91		0.372^a^	
Omega 6 (g)	8.8 ± 6.25	8.8 ± 4.82		0.961^a^	
Omega 6 / Omega 3	8.3 ± 10.11	7.2 ± 4.65		0.575^a^	
Vitamin A (µg)	1093.3 ± 749.66	1907.5 ± 2128.40		**0.019** ^**a**^	
Carotene (mg)	4.2 ± 4.41	9.3 ± 12.9		**0.016** ^**a**^	
Vitamin C (mg)	100.5 ± 69.28	118.0 ± 91.09		0.342^a^	
Vitamin E (mg)	12.3 ± 7.69	13.1 ± 6.27		0.621^a^	
Vitamin B_12_ (mg)	3.9 ± 2.52	3.0 ± 1.88		0.094^a^	
Folic acid (mg)	287.5 ± 124.08	272.4 ± 118.61		0.600^a^	
Vitamin K (mcg)	102.0 ± 108.32	116.6 ± 143.04		0.613^a^	
Potassium (mg)	2299.4 ± 795.55	2516.6 ± 1089.98		0.316^a^	
Calcium (mg)	732.3 ± 293.48	744.0 ± 309.15		0.868^a^	
Magnesium (mg)	232.7 ± 78.44	243.1 ± 91.31		0.597^a^	

^a^ Student’s t-test,

Significant correlations and the corresponding p-values are presented in bold

The consumption of red meat, white meat, and butter was determined to be statistically significantly greater in the case group than in the control group (*p* < 0.05). The consumption of fish was observed to be statistically significantly higher in the control group than in the case group (*p* < 0.05) (Table 3).

**Table 3:** Mean daily consumption of food groups

Food Groups	Case (*n* = 47)	Control (*n* = 30)	
	Mean ± SD	Mean ± SD	*p*
**Dairy products (g)**			
Milk, yogurt, ayran, kefir	178.7 ± 184.7	139.1 ± 173.62	0.350^a^
Cheese (total)	35.5 ± 20.68	38.0 ± 23.80	0.627^a^
**Meat products total (g)**			
Red meat	42.3 ± 53.71	19.4 ± 24.42	**0.032** ^**a**^
Offal	2.5 ± 9.63	3.2 ± 10.42	0.747^a^
White meat	25.0 ± 50.82	31.0 ± 69.86	0.664^a^
Fish	5.1 ± 23.40	29.8 ± 68.81	**0.026** ^**a**^
Eggs	39.9 ± 34.94	28.7 ± 30.03	0.303^a^
Dried legumes and nuts	48.5 ± 30.03	53.8 ± 59.15	0.609^a^
**Fruit-vegetable group total (g)**			
Vegetables	245.1 ± 179.65	242.8 ± 129.21	0.952^a^
Fruit	156.3 ± 167.66	155.2 ± 151.2	0.948^a^
**Grains, bread group total (g)** 232.6 ± 123.85		205.3 ± 121.3	0.345^a^
**Oil groups (g)**			
Olive oil	4.3 ± 7.49	4.9 ± 5.64	0.688^a^
Other plant oils	6.3 ± 10.65	5.2 ± 6.86	0.594^a^
Butter	5.9 ± 10.23	1.6 ± 3.18	**0.030** ^**a**^
Margarine	15.1 ± 15.07	14.8 ± 14.36	0.932^a^
**Desserts (g)**	12.6 ± 21.80	17.2 ± 23.13	0.383^a^

^a^ Student’s t-test

Significant correlations and the corresponding p-values are presented in bold

The TOC (4.6 ± 3.63 mmol Trolox Equivalent) and OSI (0.2 ± 0.05) levels of the case group were determined to be statistically significantly higher than those of the control group (3.6 ± 1.40 mmol Trolox Equivalent, and 0.2 ± 0.07) (*p* < 0.001).The TAC level (1.91 ± 0.21 µmol H_2_O_2_ Equivalent and 3.6 ± 1.40) of the case group was lower than that of the control group but not at a statistically significant level (*p* = 0.064). The DTAC was mean 10.6 ± 3.89 mmol in the case group and 12.4 ± 3.57 mmol in the control group (*p* < 0.05) (Table 4).

**Table 4:** Dietary and serum total antioxidant capacity of the groups

Parameters	Case (*n* = 47)	Control (*n* = 30)	
	Mean ± SD	Mean ± SD	*p*
Serum Total Antioxidant Capacity			
TAC (mmol Trolox Equivalent)	1.9 ± 0.21 (1.41–2.40)	2.0 ± 0.22 (1.70–2.49)	0.064^a^
TOC (µmol H_2_O_2_ Equivalent)	4.6 ± 3.63 (2.94–7.04)	3.6 ± 1.40 (2.18–8.21)	**0.001** ^**a**^
OSI (Arbitrary unit)	0.24 ± 0.05 (0.16-0.42)	0.18 ± 0.07 (0.10–0.48)	**0.000** ^**a**^
**Dietary Total Antioxidant Capacity (DTAC)**	10.6 ± 3.89 (4.63–19.97)	12.4 ± 3.57 (5.86-19.00)	**0.043** ^**a**^

^a^ Student’s t- test

TAC: Total antioxidant capacity

TOC: Total oxidant capacity

OSI: Oxidative stress index

DTAC: Dietary Total Antioxidant Capacity

Significant correlations and the corresponding p-values are presented in bold

## Discussion

The results of this study demonstrated that the majority (83%) of the patients with OA were slightly overweight or obese. In the comparisons between the OA patients and the healthy control group, serum oxidant levels in the OA group were higher and dietary antioxidant levels were lower than in the control group.

A negative correlation has been reported between obesity and the number of meals eaten with a decrease observed in body weight as the number of meals increased [29]. In addition, a slowing down of the basal metabolic rate has been associated with skipping meals. In a study in Korea (*n* = 27,220), a relationship was observed between a decrease in the number of meals and obesity and metabolic syndrome [30]. The finding in the current study that half of the patients with OA skipped main meals could be associated with the extremely high prevalence (83.0%) of being slightly overweight or obese.

Omega-3 fatty acids, which reduce bone and cartilage degeneration, prevent the expression of proinflammatory cytokines and help to inhibit ROS formation and activation of the NF-κB pathway, are extremely important in the pathogenesis and treatment of osteoarthritis [8]. In a double-blind, randomized controlled study (*n* = 202), it was reported that fish oil supplements given to OA patients reduced the pain associated with OA [31]. In a similar study, one group of patients received 1000 mg fish oil supplementation for 8 weeks, a second group received 2000 mg fish oil supplementation for 8 weeks, and a third group received no supplementation. The study results demonstrated that knee functions improved in the OA patients who had received the fish oil supplement [32]. In the current study, the consumption of red meat and white meat was observed to be greater in the OA patient group than in the control group (*p* < 0.05) (Table 3). Moreover, the control group was found to consume statistically significantly more fish (*p* < 0.05). The lower amount of fish consumed by the OA patients could have contributed to the formation of OA by reducing the dietary intake of omega-3 fatty acid.

It has been suggested that a diet rich in antioxidants could be protective against OA and therefore could reduce the incidence of the disease. As vitamins A, C, and E have antioxidant properties, they could be helpful in reducing OA symptoms [33]. However, there is no study in the literature which has solely investigated vitamin A supplementation in OA [8]. The results of the current study showed that the amounts of vitamin A and carotene consumed by the case group were significantly lower than those of the control group (*p* < 0.05) (Table 2).

It is accepted that oxidative stress increasing together with obesity triggers the formation of OA and aggravates the symptoms of the disease. Intake of antioxidants at a high amount reduces oxidative stress, and thus reduces the risk of inflammation-related diseases such as obesity and metabolic syndrome, which can cause osteoarthritis [34]. Furthermore, obesity accelerates the production of inflammatory cytokines in adipose tissue by stimulating T-cells and causing macrophage activation [35].

In a study by Altay et al. [36], oxidant/antioxidant capacity levels were compared between patients with knee OA and healthy individuals. The individuals in the case group were determined to be generally obese (30.0 ± 4.4 kg/m^2^). To be able to correctly evaluate the results the control group was formed of subjects of similar age and BMI (30.5 ± 4.5 kg/m^2^) (*p* > 0.05). In the results of the study, the serum lipid peroxide, TOC, and OSI levels of the knee OA group were determined to be significantly higher than those of the control group (*p* < 0.05). Bagherifard et al. [37] reported that the TOC and OSI values (23.3 ± 7 μm and 0.38 ± 0.09) of patients with knee OA were significantly higher than those of the control group (14.2 ± 2 μm and 0.72 ± 0.3) (*p* < 0.05). It was also shown in that study that the TAC level of the control group was higher (control group: 38.8 ± 6.6 μm; case group: 35.8 ± 12 μm) but was not determined to be statistically significant. In the current study, when the serum antioxidant/oxidant levels were examined, the TOC and OSI levels of the case group were determined to be significantly higher in the case group than in the control group (*p* < 0.001), and the TAC level was lower but not at a statistically significant level (*p* > 0.05).

The dietary total antioxidant capacity formed by the ferritin-reducing property of antioxidants is an important method used in the investigation of the role of dietary intake antioxidant nutrients and nutrient components in the formation and prevention of diseases. Amarkhizi et al. [38]. investigated DTAC and inflammatory and oxidative stress biomarkers in 160 patients with osteoarthritis. They found that DTAC scores is 12.05 ± 5.3 and inverse associations between the DTAC score and disease severity. Also their study showed that patients in the highest quartile of DTAC had lower serum levels of inflammatory factors. In a study that examined the relationship between diabetes and antioxidant nutrition, a negative correlation was observed between DTAC and Homeostatic Model Assessment Insulin Resistance (HOMA-IR) [39]. In a similar study, the DTAC and metabolic syndrome components were examined in 153 healthy individuals. That study found a positive relationship between TAC and fibre, folic acid, vitamins A and C, magnesium, selenium, and zinc intake, and a negative relationship between TAC and lipid levels. A negative correlation was also observed between BMI and TAC values [40]. In a previous study, the frequency of food consumption in individuals was obtained with the FRAP-derived non-enzymatic antioxidant capacity method and these subjects were followed up for 19 years. While no relationship was determined between the risk of OA and the dietary intake of Vitamin C, β-carotene, and dietary antioxidant capacity levels, high Vitamin C intake was found to increase the risk of OA [41]. In the current study, the amount of dietary intake of antioxidants of the case group was found to be significantly lower than that of the control group (*p* < 0.05) (Table 4). The reason for this can be attributed to the lower intake of vitamin A and carotene, and the greater amount of butter and red meat consumption by the case group than the control group.

The strengths of the present study included both dietary and serum total antioxidant capacity calculates and comparison, there is a limited number of studies in literature that have examined the relationship between total antioxidant capacity of the diet and osteoarthritis.

There were some limitations to this study. The dietary data analyzed in the study were obtained from food frequency questionnaire and 24-hour-recall methods, and therefore do not represent long-term nutritional habits. In addition, the results may have been affected by possible deviations in the basic characteristics of the included population, such as the impact of the poverty income ratio.

The study demonstrated that the patients with OA were generally overweight, ate fewer snacks than the control group, and consumed less food high in n-3 fatty acids such as fish, and ate more food high in n-6 fatty acids such as butter and red meat. The serum oxidant levels of the patients with OA were determined to be high, and the dietary total antioxidant levels were low. Therefore, menu planning of OA patients should aim for a diet rich in antioxidant vitamins and minerals. Foods containing saturated fats should be consumed less because of the inflammatory effects, and care should be taken to provide a diet with a high omega-3/omega-6 ratio.

This study showed that negative association between serum TAC and DTAC patients with OA. The findings suggest that patients with OA have lower dietary TAC and higher oxidative stress than healthy individuals. Further studies with a larger population are needed to support our study.

## Data Availability

No datasets were generated or analysed during the current study.
